# Silent and Deadly: Incidentally Discovered Asymptomatic Left Ventricular Pseudoaneurysm

**DOI:** 10.1016/j.case.2025.06.005

**Published:** 2025-10-03

**Authors:** Greg Douglas, Christopher Balfe, Mark Russell, David Moore

**Affiliations:** Department of Cardiology, Tallaght University Hospital, Dublin, Ireland

**Keywords:** Left ventricular pseudoaneurysm, Ultrasound enhancing agent, Transthoracic echocardiogram, Cardiovascular magnetic resonance imaging

## Abstract

• Asymptomatic MI can lead to formation of chronic LVP.
• Echocardiography is essential in the diagnosis of LVP.
• There is a role for nonsurgical management of chronic LVP.
• Management of chronic LVP includes anticoagulation and strict blood pressure control.

## Graphical abstract

**Figure:**
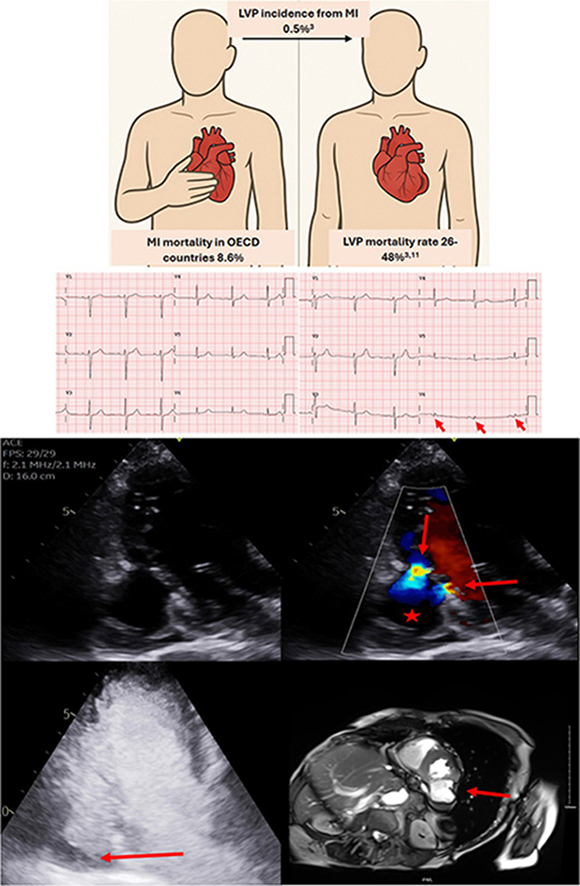

## Introduction

The management of acute myocardial infarction (MI) is constantly improving, but disparities remain, particularly when patients present atypically.^1^ Left ventricular (LV) pseudoaneurysm (LVP) is a rare but potentially life-threatening complication of myocardial free-wall rupture. It occurs when the rupture is limited by pericardium, pericardial adhesion, or thrombus, resulting in a contained outpouching from the left ventricle.^2^ Pseudoaneurysms are most associated with MI, surgery, trauma, and infection.^2^^,^^3^ We describe an intriguing case of incidental LVP detection in an elderly patient without typical cardiac symptoms.

## Case Presentation

A 78-year-old woman with no smoking history presented to her general practitioner with a chronic, productive cough over the course of 6 months. The patient had a medical history relevant for rheumatoid arthritis, osteoarthritis, and ascending aortic aneurysm. This presentation prompted a chest x-ray, which revealed a right mid-zone cavity. Following this, computed tomography (CT) of the thorax was performed, which demonstrated features of bronchiectasis. Notably, the CT thorax also revealed a 33 × 32 mm soft tissue opacity in continuity with the left ventricle.

**Figure 1:**
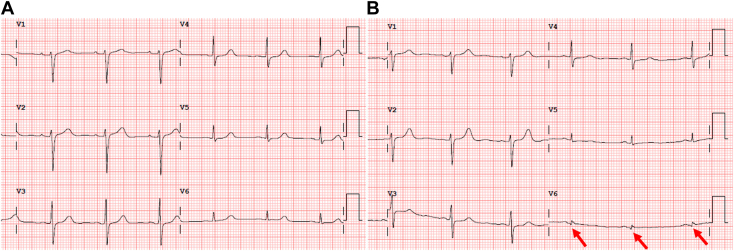
**(A)** Electrocardiogram of the patient 5 years prior to case presentation without abnormalities. **(B)** Electrocardiogram of the patient obtained after detection of the LVP, demonstrating the development of Q waves in V6 (*red arrows*).

**Figure 2:**
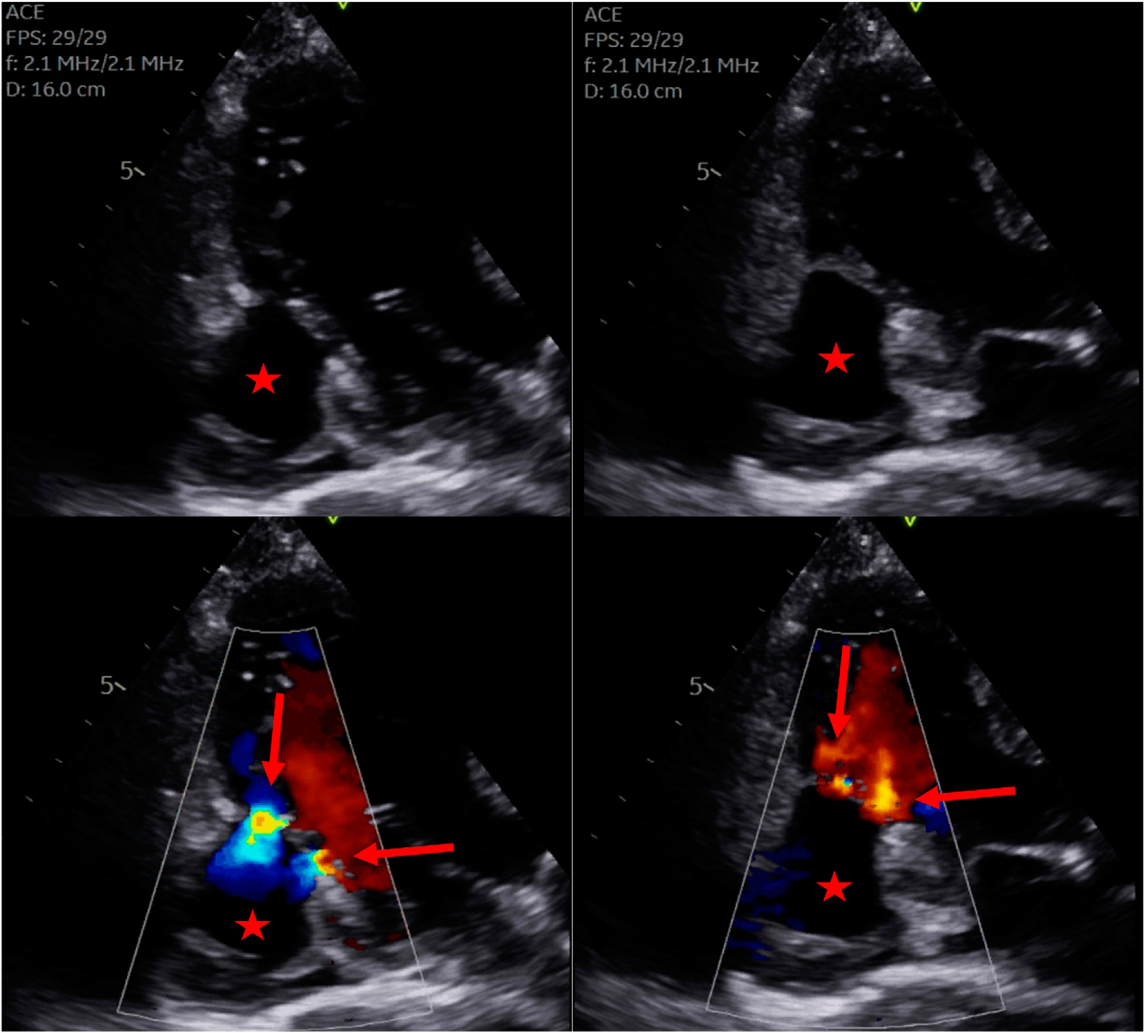
Two-dimensional TTE, apical 3-chamber diastolic (*left*) and systolic (*right*) views, without (*top*) and with (*bottom*) color-flow Doppler, demonstrates a large LVP (*star*) with thin walls in continuity with the basal and mid inferolateral LV myocardium; turbulent to-and-fro flow via 2 orifices (*arrows*) is also demonstrated.

**Figure 3:**
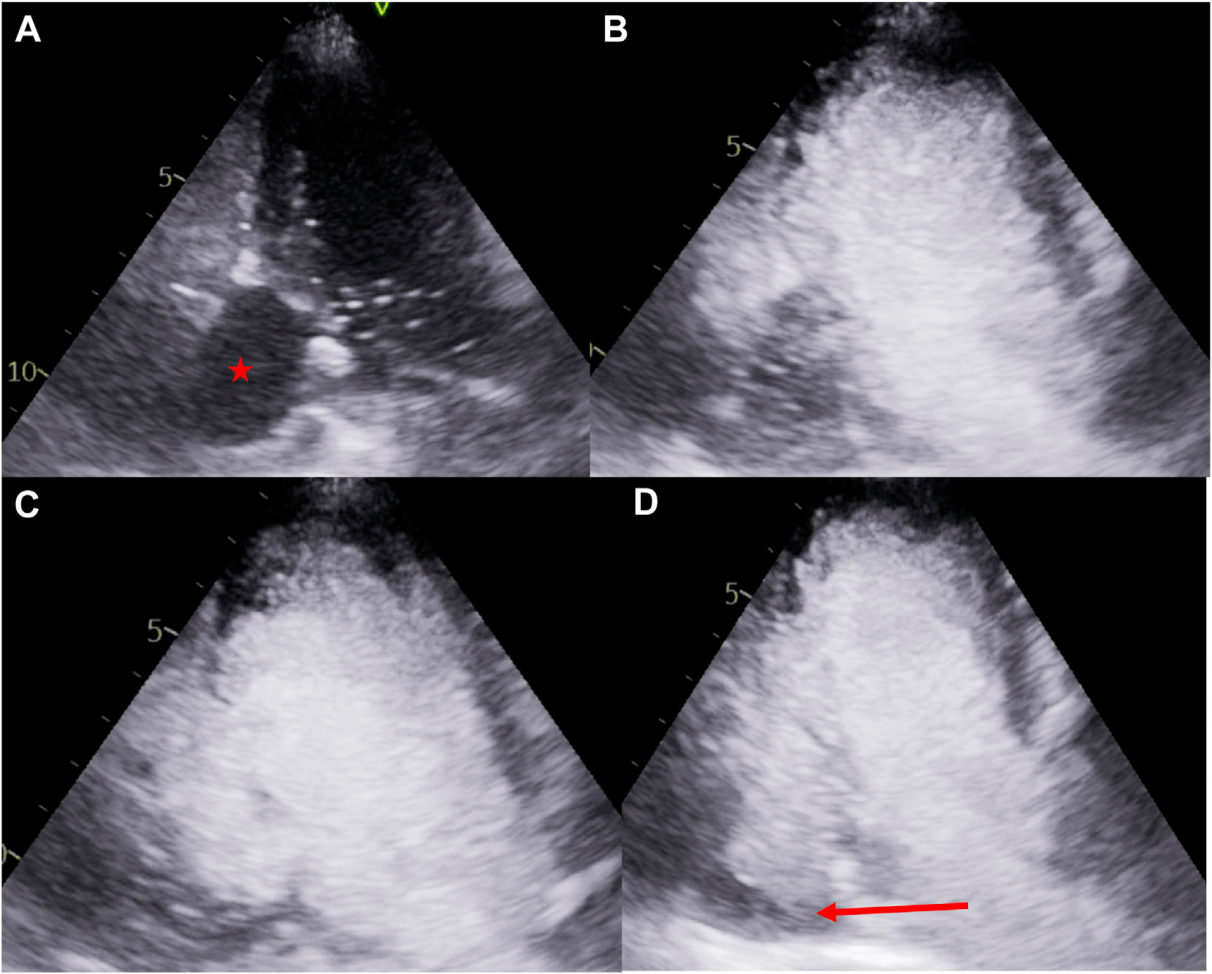
Two-dimensional TTE, apical 3-chamber view during the active administration of UEA at baseline (**A**; precontrast), early **(B)**, mid **(C),** and after complete opacification **(D)**, demonstrates the pattern and timing of flow into a large LVP (*star*); a hemispheric, linear filling defect (*arrow*) is seen along the dependent wall of the LVP consistent with a mural thrombus.

These findings led to an urgent cardiology outpatient clinic review, confirming an absence of ischemic symptoms, a normal cardiac examination, and an electrocardiogram with an abnormal Q wave in V6. This represented a new finding when compared to a prior electrocardiogram (Figure 1). This CT finding was further investigated with an urgent transthoracic echocardiogram (TTE). The initial differential diagnoses included pericardial cyst, LV aneurysm (LVA), LVP, and LV diverticulum, each considered based on imaging characteristics. The initial two-dimensional TTE demonstrated an anechoic structure adjacent to and in continuity with the inferolateral wall of the left ventricle with thin and dyskinetic walls. Color-flow Doppler was utilized to demonstrate pulsatile flow communication with the left ventricle via 2 narrow necks (Figure 2, Video 1). This was further confirmed with the administration of an ultrasound-enhancing agent (UEA), which helped refine the differential diagnosis and revealed the presence of mural thrombus (Figure 3, Video 2). Adjacent myocardial segments were attenuated and hypokinetic, suggesting that MI was the likely cause of the LVP.

**Figure 4:**
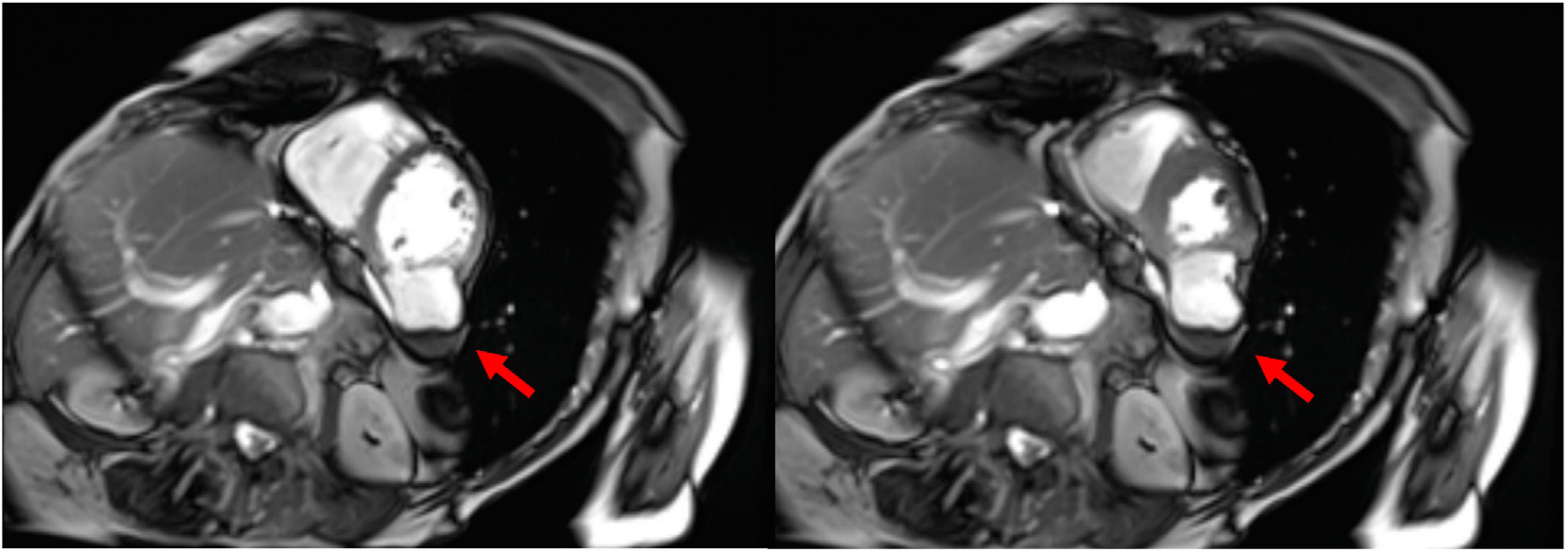
Cardiovascular magnetic resonance imaging, balanced steady state free precession (bSSFP) cine sequence, oblique axial, mid-LV short axis diastolic (*left*) and systolic (*right*) display, demonstrates normal global LV systolic function and the large LVP (*arrow*) with mural thrombus.

**Figure 5:**
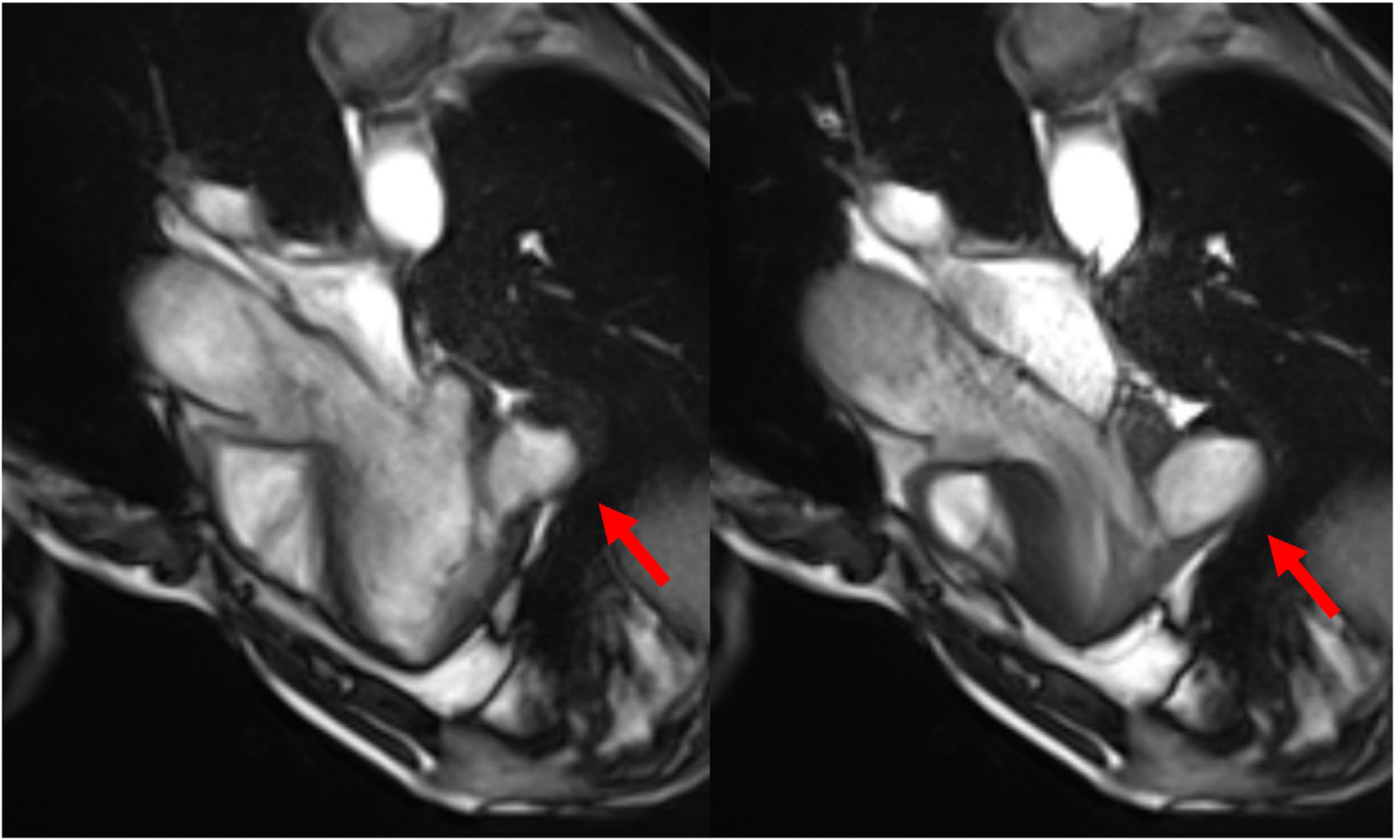
Cardiovascular magnetic resonance imaging, bSSFP cine sequence, oblique axial, long axis, 3-chamber diastolic (*left*) and systolic (*right*) display, demonstrates normal global LV systolic function and the large LVP (*arrow*).

**Figure 6:**
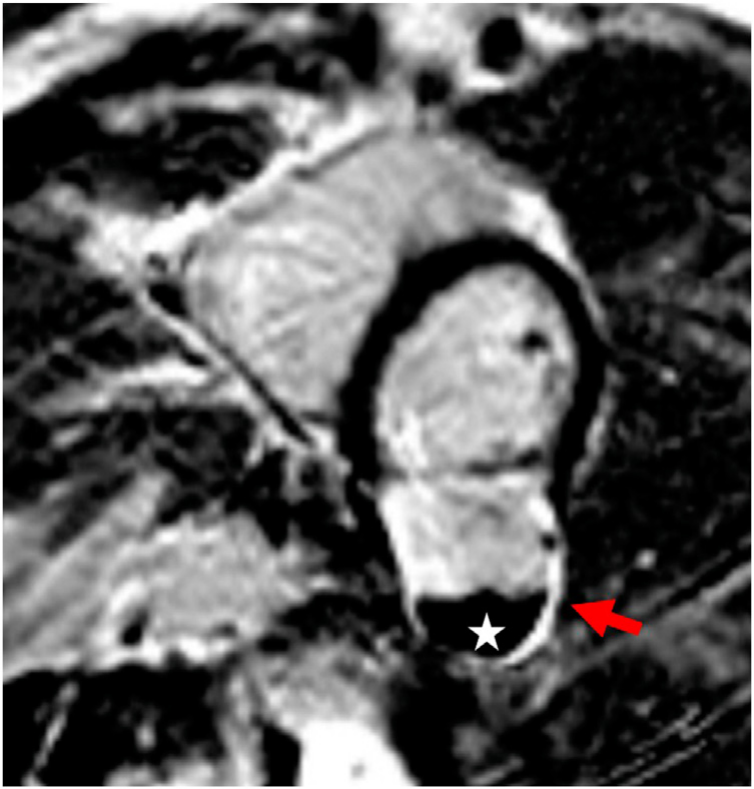
Cardiovascular magnetic resonance imaging, phase-sensitive inversion imaging, late gadolinium enhancing sequence, mid-LV short-axis display, demonstrates a markedly thinned, transmural late gadolinium enhancing inferolateral myocardial wall segment of the LVP (*arrow*) with a large, dependent nonenhancing filling defect (*star*) within its lumen consistent with thrombus.

Cardiovascular magnetic resonance imaging (CMR) provided further characterization, revealing the presence of a 4.4 × 3.6 cm noncontractile thin-walled outpouching of the inferolateral wall of the mid left ventricle, communicating via a wide neck (3.0 cm) with the left ventricle lumen (Figures 4 and 5, Videos 3 and 4). A 2.9 × 1.5 cm area of nonenhancing dependent material was observed in the outpouching, consistent with thrombus (Figure 6). The wall of the outpouching demonstrated diffuse enhancement, with very focal areas of the basal and mid inferolateral myocardium immediately adjacent to the pseudoaneurysm demonstrating abnormal transmural myocardial enhancement. These findings represent a large pseudoaneurysm with very small focal areas of transmural delayed myocardial enhancement in the LV segments immediately adjacent to the outpouching, confirming an ischemic etiology. Invasive coronary angiography was not undertaken in this case.

Following a multidisciplinary discussion between cardiologists and cardiothoracic surgeons, a consensus was reached that a nonsurgical approach would be best. This decision was based on the anatomy of the LVP, the frailty of our patient, the chronic and asymptomatic nature of the LVP, and the preserved global LV systolic function.

The decision was made to initiate the patient on anticoagulation due to the risk of embolism associated with an LVP containing thrombus. Our patient continues to be followed as an outpatient with no planned intervention and remains asymptomatic.

## Discussion

This case highlights an incidental finding of LVP without symptoms of the presumed mechanism of formation, namely, MI. Possible differential diagnoses of the initial echocardiographic findings include LVP, LVA, pericardial cyst, and LV diverticulum.

The initial identification of an anechoic structure raised the possibility of a pericardial cyst. However, communication between the left ventricle and this structure was confirmed by the presence of demonstrable flow, thus excluding that possibility.^4^

An LV diverticulum is a congenital abnormality with the characteristic of being an outpouching of the entire thickness of the myocardium. Left ventricular diverticula are differentiated from aneurysms and pseudoaneurysms by their normal contractility, usually being located at the apex.^5^ The thin, dyskinetic walls of our patient's structure went against it being an LV diverticulum.

The most challenging diagnostic distinction is between LVA and LVP. Echocardiographic indicators favoring a true aneurysm include a wide neck (ratio of neck to maximal aneurysmal diameter >0.5), anterior location, less frequent mural thrombus compared to LVP, maintained myocardial wall continuity, and absence of bidirectional flow on color-flow and pulsed-wave Doppler imaging.^3^ The TTE in this case demonstrated an anechoic structure adjacent to, and in continuity with, the inferolateral wall of the left ventricle with an abrupt discontinuity in the myocardium and 2 narrow inlets. Furthermore, color-flow Doppler demonstrated turbulent to-and-fro flow into the structure and UEA helped to highlight the presence of a mural thrombus. Taken together, these findings were in keeping with a large LVP.

Transthoracic echocardiography is the first-line imaging modality owing to it being an inexpensive and safe imaging modality. This proved to be invaluable in our case, providing the diagnosis in concert with the use of UEA. As part of the workup for surgical management, we performed a CMR to better delineate the anatomy of the LVP and quantify LV systolic function. This had the added advantage of allowing us to understand the mechanism of LVP formation, given the asymptomatic nature of the case presentation. Interestingly, while TTE suggested the LVP had 2 small necks, CMR demonstrated a single wide neck of 3.0 cm.

Although no definitive guidelines exist, some authors categorize LVP based on the time elapsed since the inciting event: acute (<2 weeks), subacute (2 weeks to 3 months), and chronic (>3 months).^6^ Acute LVPs are essentially a variant of myocardial free-wall rupture and are more likely to present with a hemodynamically unstable patient with a depressed LV systolic function. Acute LVPs also have a higher likelihood of rupture.^6^^,^^7^ Conversely, chronic LVPs present with more insidious symptoms of congestive cardiac failure, chest pain, systemic embolization, dyspnea, or arrhythmia in an otherwise stable patient. Interestingly, 10% to 48% of patients with LVP are asymptomatic.^3^^,^^8^ Given our patient's asymptomatic presentation, this case most likely represents a chronic LVP.

The distinction between acute and chronic LVP is relevant when considering the risk of rupture. Yeo *et al.*^8^ analyzed the outcomes of 52 patients with LVP and found that factors portending a greater risk of rupture included LVPs that were less than 3 months, LVPs that were larger than 3.0 cm, and anteriorly located LVPs. Additionally, LVPs associated with depressed global LV systolic function and expanding LVPs favor a surgical approach.^6^^,^^7^ Consequently, some authors advocate for surgical intervention within the first 3 months post-MI.^6^

While there are no large studies assessing outcomes of LVPs, small and asymptomatic LVPs may be managed conservatively.^6^^,^^9^^,^^10^ This is supported by the findings of Moreno *et al.*^11^ that 10% to 20% of chronic LVPs are discovered incidentally and there is a 74.1% cumulative survival at 4 years for chronic LVP treated medically. While Frances *et al.*^3^ noted a 48% mortality rate in patients managed conservatively, all of those deaths were noted to be in the acute phase, with the remaining patients surviving to 156 weeks.

The diagnosis of LVP represents a significant clinical challenge due to its rarity and often nonspecific presentation. This case highlights the importance of maintaining a high index of suspicion when assessing paracardiac masses. Although previous case reports describe incidental, asymptomatic LVPs following MI or surgery,^3^ our case is distinct in its complete lack of symptoms from both the LVP and the presumed index MI. This variability in presentation underscores the need for tailored diagnostic and management strategies. In our case, the patient’s management included anticoagulation to decrease thromboembolic risk, strict blood pressure control to decrease stress on the LVP and reduce risk of rupture,^2^^,^^3^ and ongoing follow-up to assess further symptoms or increase in size of the LVP. Despite the LVP’s large size, conservative management was favored due to the patient’s clinical stability, absence of symptoms, and preserved systolic function—factors essential in individualizing treatment decisions. This underscores the need for an individualized approach based on the patient's clinical stability and the pseudoaneurysm's characteristics.

The multimodality imaging approach utilized in this case exemplifies the strength of combining different diagnostic techniques to achieve a comprehensive assessment. The TTE provided initial insights and UEA helped to refine the diagnosis, while CMR offered detailed characterization, confirming the diagnosis and ischemic etiology. The discrepancy between TTE and CMR regarding LVP anatomy underscores the value of multimodal imaging in LVP characterization.

Although comprehensive data on LVP incidence remain limited,^9^ advancements in percutaneous coronary intervention have led to a decline in mechanical complications following MI.^5^ The rise in the utilization of advanced imaging techniques could potentially lead to an increase in the detection of chronic LVPs.^1^^,^^6^^,^^12^ This could add to the body of evidence regarding the management of this rare entity. Further research is needed to confirm this trend and establish optimal management protocols for patients with chronic LVPs.

## Conclusion

Left ventricular pseudoaneurysm represents a rare yet clinically important diagnosis, often with nonspecific and ambiguous signs, symptoms, and investigations. Multimodal imaging plays a crucial role in refining the differential diagnosis and confirming the diagnosis. Optimal management of LVP requires careful consideration of multiple factors, and this case highlights the viability of a nonsurgical approach.

## Disclosure Statement

The authors declare that there is no conflict of interest.

## Supplementary Data

Video 1 Two-dimensional TTE, apical 3-chamber view without (*left*) and with (*right*) color-flow Doppler, demonstrates normal global LV systolic function with a large LVP with thin walls in continuity with the basal and mid inferolateral LV myocardium; turbulent to-and-fro flow via 2 orifices is also demonstrated.

Video 2 Two-dimensional TTE, apical 3-chamber view during the active administration of UEA, demonstrates the pattern and timing of flow into a large LVP and a hemispheric, linear filling defect along the dependent wall of the LVP consistent with a mural thrombus.

Video 3 Cardiovascular magnetic resonance imaging, bSSFP cine sequence, oblique axial, mid-LV short axis display, demonstrates normal global LV systolic function and the large LVP (*arrow*) with mural thrombus.

Video 4 Cardiovascular magnetic resonance imaging, bSSFP cine sequence, oblique axial, long axis 3-chamber display, demonstrates normal global LV systolic function and the large LVP (*arrow*).
